# Secretoneurin A Directly Regulates the Proteome of Goldfish Radial Glial Cells *In Vitro*

**DOI:** 10.3389/fendo.2018.00068

**Published:** 2018-03-06

**Authors:** Dillon F. Da Fonte, Chris J. Martyniuk, Lei Xing, Vance L. Trudeau

**Affiliations:** ^1^Department of Biology, University of Ottawa, Ottawa, ON, Canada; ^2^Department of Physiological Sciences, College of Veterinary Medicine, UF Genetics Institute, University of Florida, Gainesville, FL, United States

**Keywords:** secretogranin II, secretoneurin, radial glial cells, aromatase, neurogenesis

## Abstract

Radial glial cells (RGCs) are the main macroglia in the teleost brain and have established roles in neurogenesis and neurosteroidogenesis. They are the only brain cell type expressing aromatase B (*cyp19a1b*), the enzyme that synthesizes estrogens from androgen precursors. There are few studies on the regulation of RGC functions, but our previous investigations demonstrated that dopamine stimulates *cyp19a1b* expression in goldfish RGCs, while secretoneurin A (SNa) inhibits the expression of this enzyme. Here, we determine the range of proteins and cellular processes responsive to SNa treatments in these steroidogenic cells. The focus here is on SNa, because this peptide is derived from selective processing of secretogranin II in magnocellular cells embedded within the RGC-rich preoptic nucleus. Primary cultures of RGCs were treated (24 h) with 10, 100, or 1,000 nM SNa. By using isobaric tagging for relative and absolute quantitation and a Hybrid Quadrupole Obritrap Mass Spectrometry system, a total of 1,363 unique proteins were identified in RGCs, and 609 proteins were significantly regulated by SNa at one or more concentrations. Proteins that showed differential expression with all three concentrations of SNa included H1 histone, glutamyl-prolyl-tRNA synthetase, Rho GDP dissociation inhibitor γ, vimentin A2, and small nuclear ribonucleoprotein-associated protein. At 10, 100, and 1,000 nM SNa, there were 5, 195, and 489 proteins that were downregulated, respectively, whereas the number of upregulated proteins were 72, 44, and 51, respectively. Subnetwork enrichment analysis of differentially regulated proteins revealed that processes such as actin organization, cytoskeleton organization and biogenesis, apoptosis, mRNA processing, RNA splicing, translation, cell growth, and proliferation are regulated by SNa based on the proteomic response. Moreover, we observed that, at the low concentration of SNa, there was an increase in the abundance of proteins involved in cell growth, proliferation, and migration, whereas higher concentration of SNa appeared to downregulate proteins involved in these processes, indicating a dose-dependent proteome response. At the highest concentration of SNa, proteins linked to the etiology of diseases of the central nervous system (brain injuries, Alzheimer disease, Parkinson’s disease, cerebral infraction, brain ischemia) were also differentially regulated. These data implicate SNa in the control of cell proliferation and neurogenesis.

## Introduction

Radial glial cells (RGCs) are a macroglial subtype present during central nervous system (CNS) development of all vertebrates and are characterized by their bipolar morphology (1) and stem-like progenitor properties (2). The cell bodies of radial glia line the brain ventricles and have an elongated radial fiber terminating on the walls of blood vessels or at the pial surface with end feet (3). Due to this unique morphology, their radial processes are used as scaffolds for the migration of newborn neurons (4, 5). As stem-like cells, RGCs are capable of undergoing neurogenesis and/or gliogenesis to produce neurons or other glial cells (6). RGC populations are transient in mammals as they differentiate into astrocytes at the end of cortical development. In contrast, RGCs are abundant throughout the adult brain in teleost fish, supporting the ability for unsurpassed high levels of neurogenesis and regeneration (7, 8). In addition, teleost RGCs express various steroidogenic enzymes (9, 10) and are the exclusive cell type to express the estrogen-synthesizing enzyme, aromatase B (*cyp19a1b*) (11–13), and thus, they are neuroendocrine cells that produce neuroestrogens and other steroids in the CNS. Although RGCs in fish have established roles in neurogenesis and neurosteroidogenesis, little is known about other functions of these cells and the factors that regulate them. As RGCs are the main macroglia in fish CNS (14), they share close interactions with different neurons and can express neurotransmitters, neuropeptides, and hormone receptors (15). Therefore, these cells may be under functional control through neuronal–glial interactions.

We previously reported on the close neuroanatomical relationship between the soma of secretoneurin A (SNa)-immunoreactive magnocellular preoptic neurons and RGCs along the third ventricle in goldfish (16). The neuropeptide SN is generated from endoproteolytic processing of its precursor protein secretogranin II (SgII) and has well-established functions in endocrine, nervous, and immune systems (17, 18). Currently, there are two known SgII paralogs, SgIIa and SgIIb, that likely arose from the genome duplication events in teleost fish, and these paralogs produce SNa and SNb peptides, respectively (19). Both *in vivo* and *in vitro*, SNa reduces *cyp19a1b* mRNA levels in goldfish RGCs, implicating SNa in the control of neuroestrogen production (16). Furthermore, data from transcriptome sequencing of cultured goldfish RGCs revealed that gene networks related to immune responses and CNS physiology were responsive to 1,000 nM SNa (15). Altogether, these anatomical and transcriptomic data propose that SNa is a regulator of RGC functions. The objectives of this study were to (1) characterize the RGC proteome, (2) determine the differentially regulated proteins over a range of SNa concentrations, and (3) identify protein networks responsive to SNa regulation.

## Materials and Methods

### Experimental Animals

All procedures were approved by the University of Ottawa Protocol Review Committee and followed standard Canadian Council on Animal Care guidelines on the use of animals in research. Common adult female goldfish (*Carassius auratus*) were purchased from a commercial supplier (Mt. Parnell Fisheries Inc., Mercersburg, PA, USA) and allowed to acclimate for at least 3 weeks prior to experimentation. Goldfish were maintained at 18°C under a stimulated natural photoperiod and fed standard flaked goldfish food. Sexually mature female goldfish (18–35 g) were anesthetized using 3-aminobenzoic acid ethyl ester (MS222) for all handling and dissection procedures.

### Cell Culture and Exposure

The direct effects of SNa on RGCs were tested using a previously established and validated cell culture method (20). In short, the hypothalamus and telencephalon were dissected from female goldfish and rinsed twice with Hanks Balanced Salt Solution (HBSS; 400 mg KCl, 600 mg KH_2_PO_4_, 350 mg NaHCO_3_, 8 g NaCl, 48 mg Na_2_HPO_4_, and 1 g d-Glucose in 1 L ddH_2_O) with Antibiotic-Antimycotic solution (Gibco) and minced into small explants. RGCs were dissociated with trypsin (0.25%; Gibco) and cultured in Leibovitz’s L-15 medium (Gibco) with 15% fetal bovine serum (Gibco) and Antibiotic-Antimycotic solution. Cell culture medium was changed 4–7 days after isolation and then once a week thereafter. RGCs were subcultured by trypsinization (0.125%) for three passages and then used for experimentation. Goldfish SNa was synthesized as reported previously (21). Stock solutions of synthetic goldfish SNa peptide were made in water and stored at −20°C until use. Aliquots were thawed on ice then diluted to desired concentrations in serum-free media. Cells were exposed for 24 h to one of the three concentrations of goldfish SNa or the water control.

### Protein Extraction, Isobaric Tagging for Relative and Absolute Quantitation (iTRAQ), and LC-MS/MS

Proteins were extracted as previously described (22) prior to labeling and dissolved in denaturant buffer [0.1% SDS (w/v)] and dissolution buffer (0.5 M triethylammonium bicarbonate, pH 8.5) in the iTRAQ Reagents 8-plex kit (AB Sciex Inc., Foster City, CA, USA). For each sample, 100 µg of protein were reduced, alkylated, trypsin-digested, and labeled according to the manufacturer’s instructions (AB Sciex Inc.). Two 8-plex iTRAQ labeling reactions were conducted, each with two biological replicates. Peptides from the control group were labeled with either iTRAQ tags 113 or 114. The three treatments were labeled as follows: 10 nM SNa (115, 116), 100 nM SNa (117, 118), and 1,000 nM SNa (119, 121). This labeling scheme was repeated in a second experiment, so the data are derived from a sample size of *n* = 4 biological replicates per group.

Labeled peptides were combined for each iTRAQ experiment, desalted with C18-solid phase extraction, and dissolved in strong cation exchange solvent A [25% (v/v) acetonitrile, 10 mM ammonium formate, and 0.1% (v/v) formic acid, pH 2.8]. The peptides were fractionated using an Agilent HPLC 1260 with a polysulfoethyl A column (2.1 mm × 100 mm, 5 µm, 300 Å; PolyLC, Columbia, MD, USA). Peptides were eluted with a linear gradient of 0–20% solvent B [2 5% (v/v) acetonitrile and 500 mM ammonium formate, pH 6.8] over 50 min., followed by ramping up to 100% solvent B in 5 min. The absorbance at 280 nm was monitored, and a total of 14 fractions were collected. The fractions were lyophilized and resuspended in LC solvent A [0.1% formic acid in 97% water (v/v), 3% acetonitrile (v/v)]. A hybrid quadrupole Orbitrap (Q Exactive) MS system (Thermo Fisher Scientific) was used with high-energy collision dissociation in each MS and MS/MS cycle. The MS system was interfaced with an automated Easy-nLC 1000 system (Thermo Fisher Scientific). Each sample fraction was loaded onto an Acclaim Pepmap 100 pre-column (20 mm × 75 µm; 3 µm-C18) and separated using a PepMap RSLC analytical column (250 mm × 75 µm; 2 µm-C18) at a flow rate at 350 nL/min during a linear gradient from solvent A [0.1% formic acid (v/v)] to 25% solvent B [0.1% formic acid (v/v) and 99.9% acetonitrile (v/v)] for 80 min and to 100% solvent B for an additional 15 min.

The raw MS/MS data files were processed by a thorough database searching approach considering biological modification and amino acid substitution against the National Center for Biotechnology Information Cyprinidae database (downloaded on March 23, 2016) using the ProteinPilot v4.5 with the Fraglet and Taglet searches under ParagonTM algorithm (23). The following parameters were considered for all the searching: fixed modification of methylmethane thiosulfonate-labeled cysteine, fixed iTRAQ modification of amine groups in the *N*-terminus, lysine, and variable iTRAQ modifications of tyrosine. The MS/MS spectra that (1) were unique to a specific protein and (2) showed a sum of the signal-to-noise ratios for all the peak pairs greater than 9 were used for quantification. A protein was quantified if it was represented with at least three unique spectra in at least two of the biological replicates, along with a Fisher’s combined probability of <0.05 and a fold change of <0.5 or >1.5.

### Gene Ontology (GO) and Pathway Analysis

Gene ontology terms were assigned using protein annotation through evolutionary relationships to classify all proteins identified in RGC cultures (24). GO categories for biological processes, molecular functions, cellular components, protein classes, and pathways were used to identify the distribution of proteins within each GO category. Pathway Studio 9.0 (Elsevier) and ResNet 10.0 were used to build a protein interaction network for SNa effects in RGCs. Official gene symbols were manually retrieved using Gene Cards (http://www.genecards.org) to map proteins into Pathway Studio. The number of proteins that successfully mapped to Pathway Studios using Name + Alias was as follows: 10 nM SNa = 99, 100 nM SNa = 231, and 1,000 nM SNa = 499. Interaction networks were based on expression, binding, and regulatory interactions in the database and were constructed using direct connections with one neighbor. Subnetwork enrichment analysis (SNEA) for cell processes was performed in Pathway Studio to determine if differentially expressed proteins were related to specific biological functions. *P* value for gene seeds was set at < 0.05 and the criterion of >5 members per cell process or group was required for inclusion as a significantly regulated gene network.

## Results

### Identification and GO Classification of RGC Proteins

**Figure 1:**
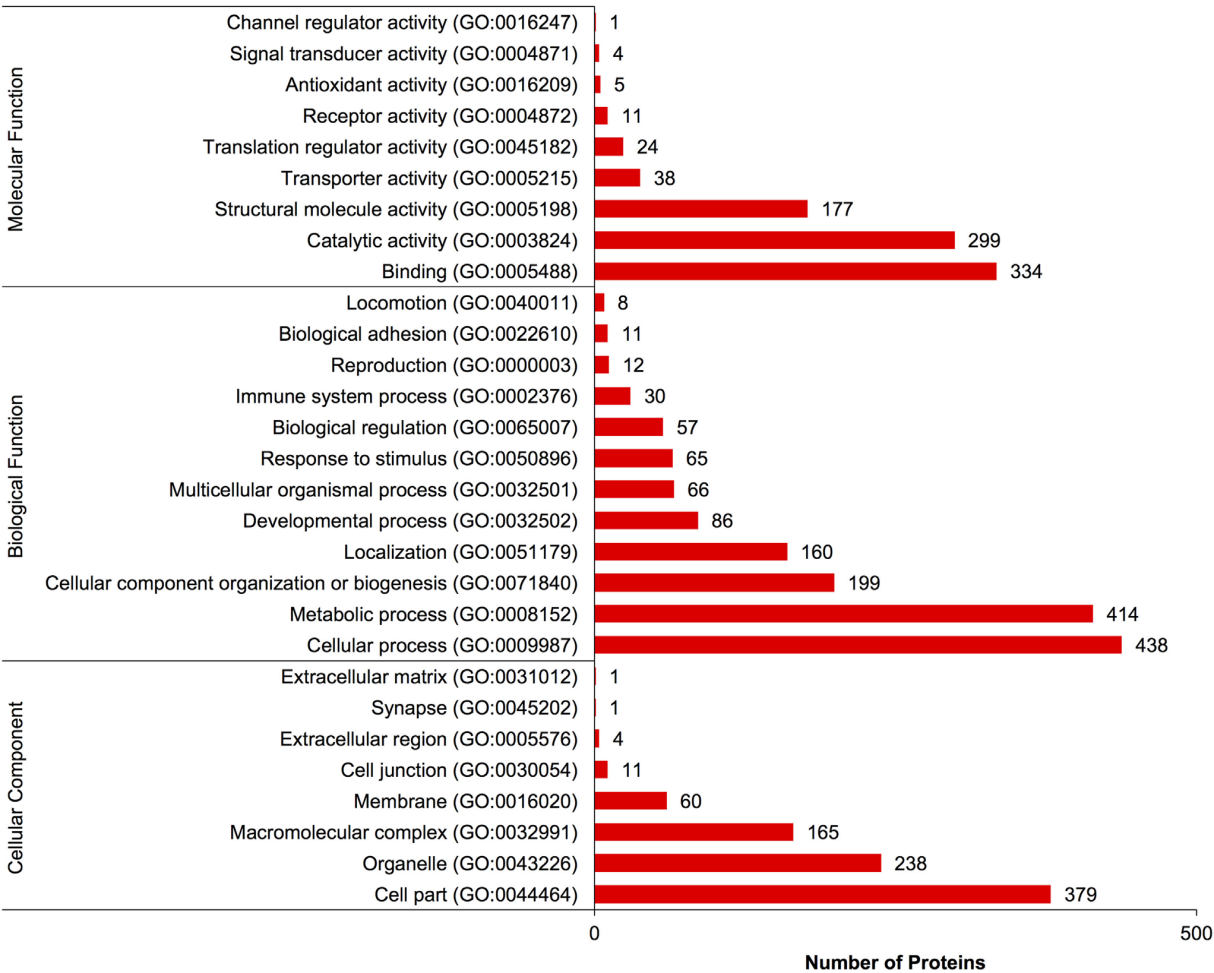
Gene ontology classification of *Carassius auratus* radial glial cell (RGC) proteins into molecular function, biological function, and cellular component categories. The number of proteins ascribed to each classification, along with accession number, is provided.

**Figure 2:**
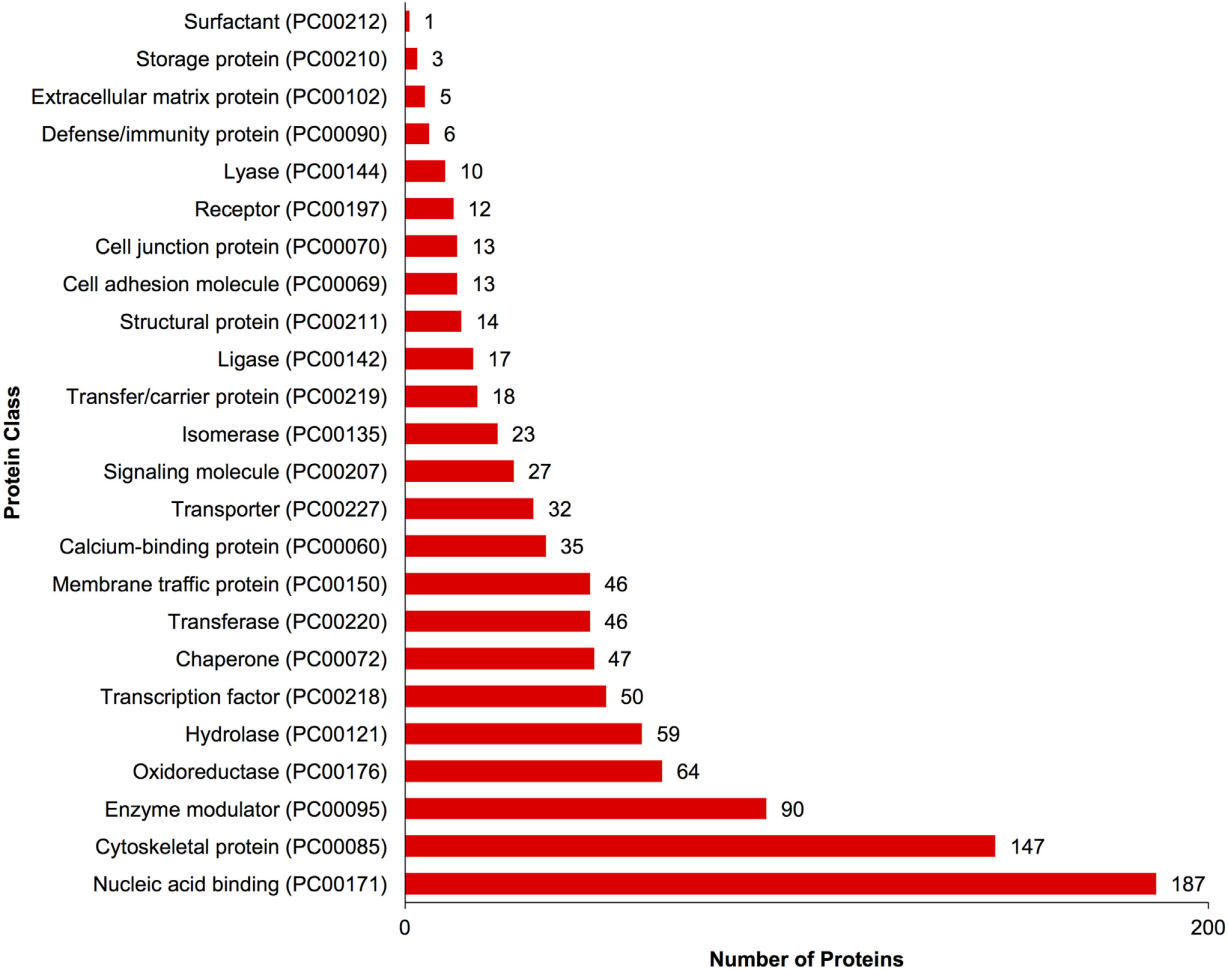
Gene ontology classification of *Carassius auratus* radial glial cell (RGC) proteins by protein class. The number of proteins ascribed to each classification, along with accession number, is provided.

A complete list of proteins (*n* = 1,363) that were identified in this study is provided in Table S1 in Supplementary Material. The annotated proteins were assigned GO terms and classified in three main GO categories: biological function, molecular function, and cellular component (Figure 1). A variety of biological functions were identified with 1,546 unique biological processes classified into 12 functional groups. The most represented biological functions included the categories of cellular process (438 proteins), metabolic process (414), and cellular component organization or biogenesis (199). Other important biological function allocations included the categories of developmental process (86 proteins), response to stimulus (65), immune system process (30), and biological adhesion (11). A broad array of 893 molecular functions was categorized into 9 functional groups. The molecular functions most represented in the RGC proteome were binding (334 proteins), catalytic (299), structural molecule (177), and transporter (38) activities. When organized by cellular component, RGC proteins were enriched in the categories of cell part (379 proteins), organelle (238), and macromolecular complex (165). Most proteins identified in RGCs were further classified into protein class, with nucleic acid binding (187 proteins), cytoskeletal proteins (147), and enzyme molecular (90) being the most represented classes detected in RGCs (Figure 2). Finally, a total of 535 proteins were represented by 102 pathways. The top 25 represented pathway ontologies are shown in Table 1. Proteins were most enriched in relation to Parkinson disease (31 proteins), integrin signaling pathway (28), ubiquitin proteasome pathway (26), cytoskeletal regulation by Rho GTPase (26), Huntington disease (25), and inflammation mediate by chemokine and cytokine signaling (24) (Table 1). Comparison of the assigned pathway ontologies identified by transcriptomics (15) and proteomics reveals high similarity. For example, of the top 25 pathways from transcriptomic and proteomic datasets, 18 (72%) were identical (Table 1). A full list of GO classifications can be found in Table S2 in Supplementary Material.

**Table 1:** Top 25 pathway ontologies associated with *Carassius auratus* RGC proteins based on number of protein identified in each pathway.

Pathway name	Pathway accession	# of proteins identified
Parkinson disease^a^	P00049	31
Integrin signaling pathway^a^	P00034	28
Ubiquitin proteasome pathway	P00060	26
Cytoskeletal regulation by Rho GTPase^a^	P00016	26
Huntington disease^a^	P00029	25
Inflammation mediated by chemokine and cytokine signaling pathway^a^	P00031	24
FGF signaling pathway^a^	P00021	15
EGF receptor signaling pathway^a^	P00018	15
CCKR signaling map^a^	P06959	14
Wnt signaling pathway^a^	P00057	14
Angiogenesis^a^	P00005	13
Gonadotropin-releasing hormone receptor pathway^a^	P06664	12
Cadherin signaling pathway^a^	P00012	12
Alzheimer disease-presenilin pathway^a^	P00004	10
T cell activation^a^	P00053	10
Nicotinic acetylcholine receptor signaling pathway	P00044	10
Ras pathway^a^	P04393	10
Glycolysis	P00024	10
Cell cycle	P00013	10
Apoptosis signaling pathway^a^	P00006	8
TGF-beta signaling pathway^a^	P00052	7
*De novo* purine biosynthesis	P02738	7
PDGF signaling pathway^a^	P00047	7
Dopamine receptor-mediated signaling pathway	P05912	7
Axon guidance mediated by Slit/Robo	P00008	6

*^a^These pathways were also identified in the *de novo* transcriptome assembly data in Ref. (15)*.

### Quantitative Proteomic Responses in Radial Glia to SNa *In Vitro*

**Figure 3:**
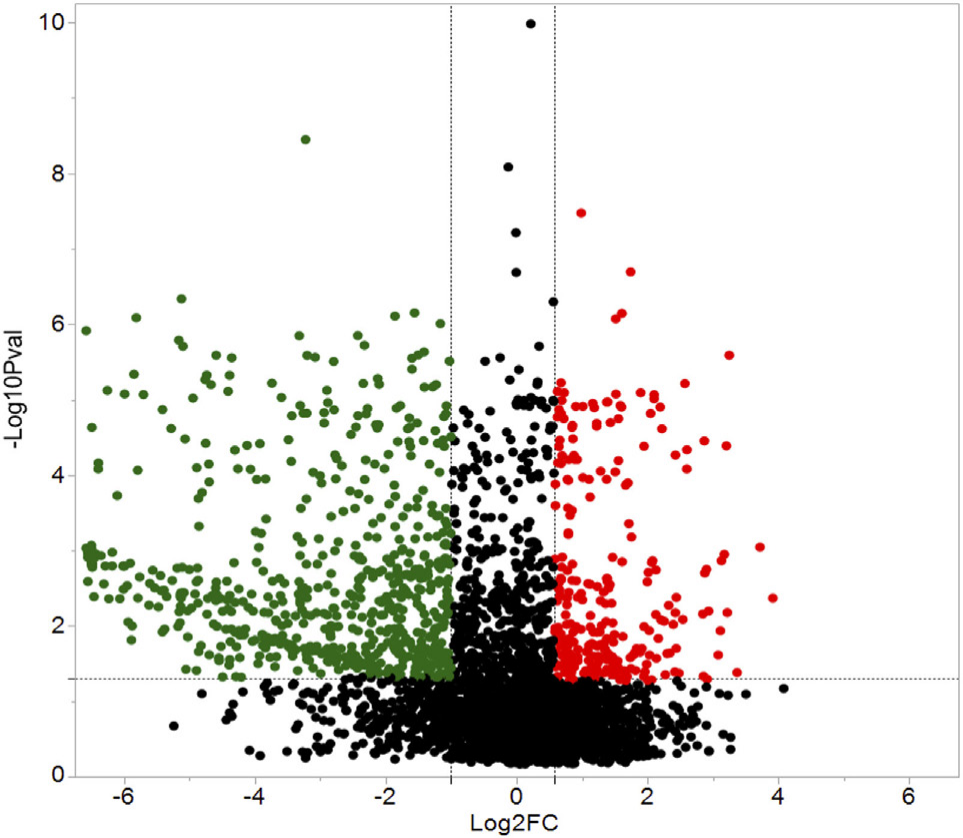
Volcano plots for protein expression in *Carassius auratus* radial glial cells (RGCs) treated with three concentrations of secretoneurin A. Significantly regulated proteins were determined using a cut off of >1.5 or <0.5 and *P* < 0.05. Red and green dots represent proteins that are upregulated and downregulated, respectively.

**Figure 4:**
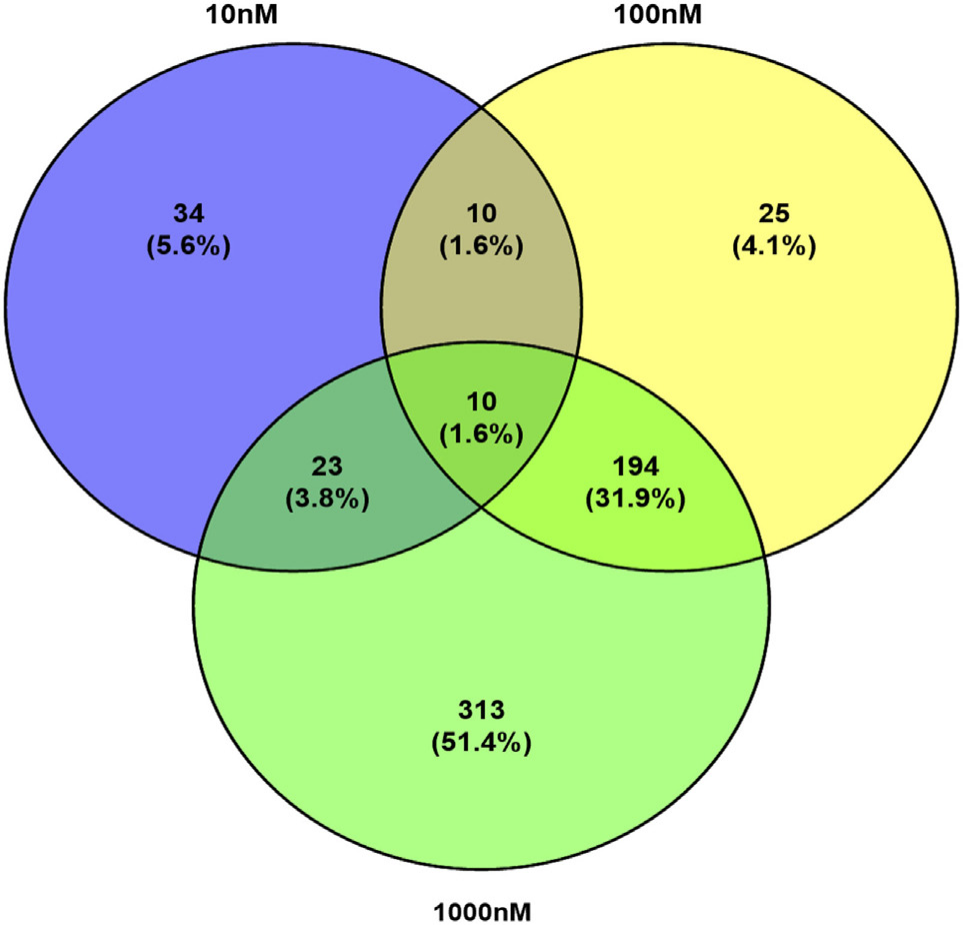
Venn diagram showing the overlap of differentially expressed proteins following secretoneurin A (10–1,000 nM) treatment in *Carassius auratus* radial glial cells.

A total of 609 proteins differed in abundance following SNa treatment (Figure 3), and there were 17 unique expression patterns recognized within the data set (Table 2). At 10, 100, and 1,000 nM SNa, there were 5, 195, and 489 downregulated proteins, respectively, whereas the numbers of upregulated proteins were 72, 44, and 51, respectively. There were more downregulated proteins observed as the concentration of SNa increased in the cultures. The overlap of the differentially regulated proteins between concentrations is shown in Figure 4. A total of 10 common proteins were regulated in all three concentrations, and some examples include H1 histone, glutamyl-prolyl-tRNA synthetase, Rho GDP dissociation inhibitor γ, vimentin A2, and small nuclear ribonucleoprotein-associated protein. Table 3 highlights the top 20 proteins that were most regulated by each concentration of SNa based on the *P* value.

**Table 2:** Expression patterns of the differentially regulated proteins in *Carassius auratus* RGCs treated with various concentrations of SNa compared to control.

Expression pattern	10 nM	100 nM	1000 nM	Number of protein IDs
I	↑	↑	↑	5
II	↑	↑	–	9
III	↑	–	↑	3
IV	↑	–	–	31
V	↑	–	↓	20
VI	↑	↓	↓	4
VII	–	↑	↑	11
VIII	–	↑	–	18
IX	–	↑	↓	1
X	–	–	↑	25
XI	–	–	–	757
XII	–	–	↓	288
XIII	–	↓	↑	6
XIV	–	↓	–	7
XV	–	↓	↓	176
XVI	↓	–	–	3
XVII	↓	↓	↑	1
XVIII	↓	↓	–	1

*RGC, radial glial cell*.

**Table 3:** Top 20 proteins identified by iTRAQ as different in abundance among groups in *Carassius auratus* RGCs treated with one of three concentrations of SNa.

Concentration (nM)	Accession	Protein	% coverage protein ID	Fold change	*P* value
10	A7MCL7_DANRE	Cystatin 14a, tandem duplicate 2	47	3.34	2.02E−07
	Q6DN21_CARAU	Calmodulin long form	99.33	5.94	6.11E−06
	A0A0C5Q0E9_MEGAM	Fibroblast growth factor receptor 1b	11.62	1.54	7.76E−06
	E7F1X7_DANRE	Serine and arginine repetitive matrix 1	33.69	1.65	8.06E−06
	VIM2_CARAU	Vimentin A2	44.03	3.72	8.07E−06
	F1QNJ3_DANRE	Testin LIM domain protein	25.51	4.29	8.58E−06
	F1Q5X0_DANRE	Synaptosome associated protein 29 kDa	44.74	4.29	9.73E−06
	B0UXN7_DANRE	C-Abl oncogene 2, non-receptor tyrosine kinase	20.18	3.00	1.19E−05
	C0LYZ3_9TELE	High-mobility group box 1	55.44	2.02	1.23E−05
	A0A0R4IMX7_DANRE	SH3 and PX domains 2B	25.68	1.87	1.23E−05
	X1WGZ7_DANRE	Glutamyl-prolyl-tRNA synthetase	25.03	4.57	1.25E−05
	E7F354_DANRE	Tyrosine 3-monooxygenase/tryptophan 5-monooxygenase	57.89	3.03	1.25E−05
	Q802W6_DANRE	Arhgdia protein	39.41	2.70	2.01E−05
	Q6Y3R4_CARAU	H1 histone	34.03	4.66	2.43E−05
	B3DGP9_DANRE	Protein tyrosine kinase 2aa	11.29	1.58	3.44E−05
	Q6NYA1_DANRE	Heterogeneous nuclear ribonucleoprotein A/Bb	36.25	7.30	3.53E−05
	Q802Y1_DANRE	Serine/arginine-rich splicing factor 4	39.34	1.57	4.18E−05
	143BA_DANRE	Tyrosine 3-monooxygenase/tryptophan 5-monooxygenase activation protein beta	77.46	0.15	6.06E−05
	RBM8A_DANRE	RNA-binding protein 8A	22.99	1.90	6.22E−05
	F1QG80_DANRE	Ribosomal protein L22	34.75	1.60	7.03E−05

100	Q5U7N6_DANRE	Talin 1	18.95	0.45	9.82E−07
	Q4U0S2_DANRE	Myosin, heavy chain 11a, smooth muscle	24.77	0.49	3.08E−06
	E7FEK9_DANRE	Golgin A4	40.17	0.04	4.72E−06
	F1QIN6_DANRE	CAP-GLY domain containing linker protein 2	43.21	0.05	4.76E−06
	Q1LXT2_DANRE	Eukaryotic translation elongation factor 2a, tandem duplicate 1	26.11	1.60	5.96E−06
	F1QTN7_DANRE	Acidic leucine-rich nuclear phosphoprotein 32 family member A	27.38	0.13	7.51E−06
	E7EYW2_DANRE	Epsin 2	16.27	0.02	8.56E−06
	Q7ZW39_DANRE	Phosphoribosyl pyrophosphate synthetase 1A	22.33	0.08	9.34E−06
	F1R1J9_DANRE	AHNAK nucleoprotein	53.03	2.60	1.08E−05
	Q502F6_DANRE	Zgc:112271 protein	75.58	0.29	1.20E−05
	EIF3L_DANRE	Eukaryotic translation initiation factor 3 subunit L	22.57	0.28	1.30E−05
	C0LYZ3_9TELE	High-mobility group box 1	55.44	1.57	1.35E−05
	Q7ZU46_DANRE	Heat shock protein 4a	28.45	0.14	1.36E−05
	Q0PWB8_DANRE	PDZ and LIM domain 3b	32.7	0.11	1.51E−05
	Q9DF20_DANRE	Fragile X mental retardation 1	40.77	0.10	1.51E−05
	A4FUN5_DANRE	ISY1 splicing factor homolog	57.54	0.32	1.75E−05
	Q1LYC9_DANRE	Calpain, small subunit 1 a	47.69	0.35	2.04E−05
	Q6DRC1_DANRE	Small nuclear ribonucleoprotein F	48.84	0.17	2.90E−05
	F1QYM4_DANRE	Eukaryotic translation initiation factor 4 h	55.08	0.50	3.11E−05
	F1Q7S0_DANRE	Vesicle transport through interaction with t-SNAREs 1A	41.94	0.03	3.32E−05

1,000	Q5U7N6_DANRE	Talin 1	18.95	0.11	3.56E−09
	A0JMJ1_DANRE	Scinderin like a	33.06	0.03	4.60E−07
	Q0GC55_CARAU	Heat shock protein 47 kDa	24.81	0.34	7.06E−07
	Q6PBR5_DANRE	ATPase, H+ transporting, V1 subunit G isoform 1	80.51	3.05	7.17E−07
	F1RDG4_DANRE	si:dkey-222f2.1	55.02	0.28	7.79E−07
	Q7ZTZ6_DANRE	STIP1 homology and U-Box containing protein 1	34.51	0.02	8.19E−07
	F1R1J9_DANRE	AHNAK nucleoprotein	53.03	2.86	8.48E−07
	Q804W1_DANRE	Parvalbumin isoform 4b	45.87	0.01	1.22E−06
	F1QFN1_DANRE	ELKS/RAB6-interacting/CAST family member 1b	31.20	0.19	1.41E−06
	EIF3L_DANRE	Eukaryotic translation initiation factor 3 subunit L	22.57	0.10	1.42E−06
	Q7SXA1_DANRE	Ribosomal protein L26	55.17	0.03	1.63E−06
	E7F049_DANRE	Kinectin 1	26.47	0.20	1.90E−06
	Q7ZW39_DANRE	Phosphoribosyl pyrophosphate synthetase 1A	22.33	0.03	1.96E−06
	Q803A9_DANRE	DnaJ (Hsp40) homolog, subfamily B, member 11	11.94	0.38	2.32E−06
	A0A0R4I9C6_DANRE	Ubiquitin-fold modifier 1	73.33	0.35	2.55E−06
	B8JJS6_DANRE	Programmed cell death protein 10-B	32.86	9.51	2.58E−06
	F1QTN7_DANRE	Acidic leucine-rich nuclear phosphoprotein 32 family member A	27.38	0.04	2.58E−06
	A4FUN5_DANRE	ISY1 splicing factor homolog	57.54	0.11	2.59E−06
	Q502F6_DANRE	zgc:112271	75.58	0.12	2.74E−06
	Q9W792_DANRE	T-complex polypeptide 1	26.98	0.05	2.78E−06

*All fold changes are relative to control*.

*iTRAQ, isobaric tagging for relative and absolute quantitation; RGC, Radial glial cell; SNa, secretoneurin A*.

### Identifying Cellular Processes Affected by SNa Using SNEA

**Figure 5:**
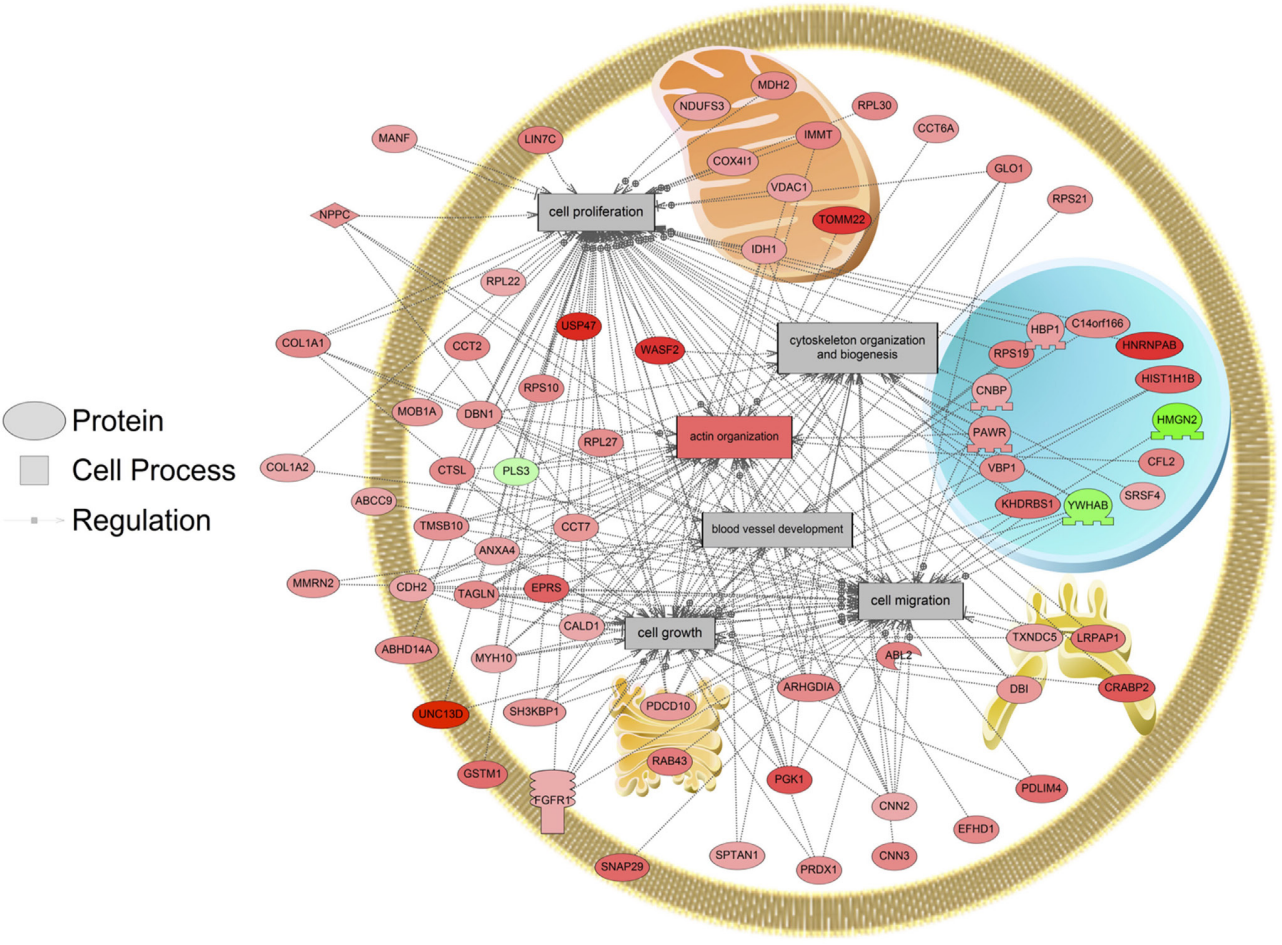
Cellular processes of blood vessel development, actin organization, cytoskeleton organization and biogenesis, cell proliferation, growth, and migration were enriched in primary *Carassius auratus* radial glial cell culture after 10 nM secretoneurin A treatment (*P* < 0.05) based on the protein profiles. Red indicates that the protein is increased, and green indicates that the protein is decreased in abundance relative to the control group. All abbreviations are provided in Table S4 in Supplementary Material.

**Figure 6:**
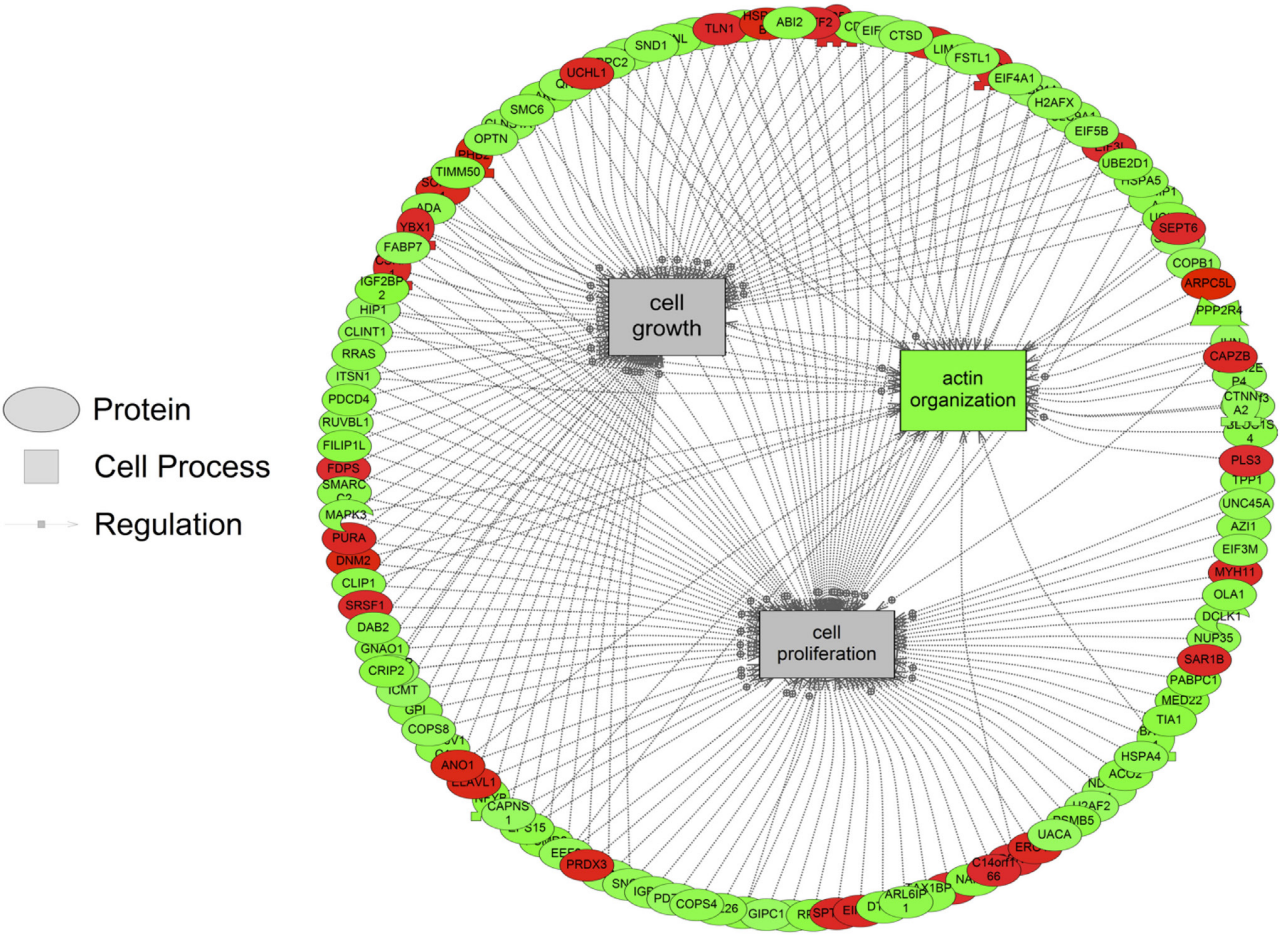
Cellular processes of actin organization, cell proliferation, and growth were significantly enriched in primary *Carassius auratus* radial glial cell culture after 100 nM secretoneurin A treatment (*P* < 0.05) based on the protein profiles. Red indicates that the protein is increased, and green indicates that the protein is decreased in abundance relative to the control group. All abbreviations are provided in Table S4 in Supplementary Material.

**Figure 7:**
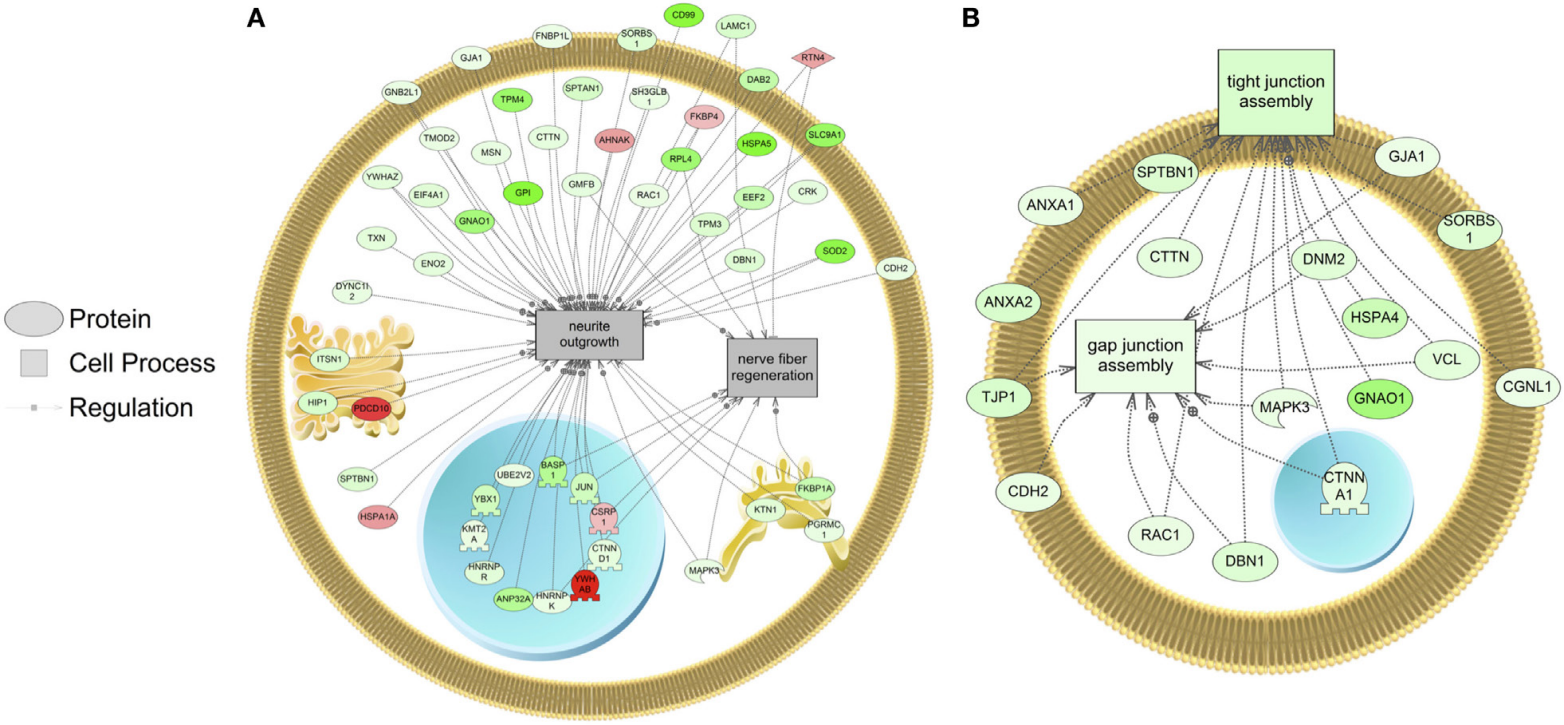
Cellular processes of **(A)** neurite outgrowth and nerve fiber regeneration and **(B)** tight and gap junction assembly were enriched in primary *Carassius auratus* radial glial cell culture after 1,000 nM secretoneurin A treatment (*P* < 0.05) based on the protein profiles. Red indicates that the protein is increased, and green indicates that the protein is decreased in abundance relative to the control group. All abbreviations are provided in Table S4 in Supplementary Material.

The major objective of this study is to identify cellular processes that were responsive to SNa through the analysis of significantly regulated RGC proteins. Processes that were regulated by all concentrations of SNa included actin organization, cytoskeleton organization and biogenesis, apoptosis, mRNA processing, RNA splicing, translation, cell growth, and proliferation. At the low 10 nM SNa dose, proteins that were increased are related to processes such as blood vessel development, actin organization, cytoskeleton organization and biogenesis, cell proliferation, growth, and migration (Figure 5). The proteins affected by 100 nM SNa control similar processes such as cell proliferation and growth; however, proteins involved in these processes were downregulated compared to 10 nM SNa (Figure 6.). Similarly, 1,000 nM SNa treatment resulted in the downregulation of proteins related to cell processes that include actin organization, cytoskeleton organization and biogenesis, cell proliferation, growth, and migration. In addition, protein responses at 1,000 nM SNa suggested enrichment of processes related to neurite outgrowth and nerve fiber regeneration (Figure 7A) and tight and gap junction assembly (Figure 7B). Both 100 and 1,000 nM SNa caused changes in proteins involved in cell cycle, mitochondrial function (mitochondrial membrane permeability, depolarization, damage), and RNA processing (RNA metabolism, processing, splicing).

**Figure 8:**
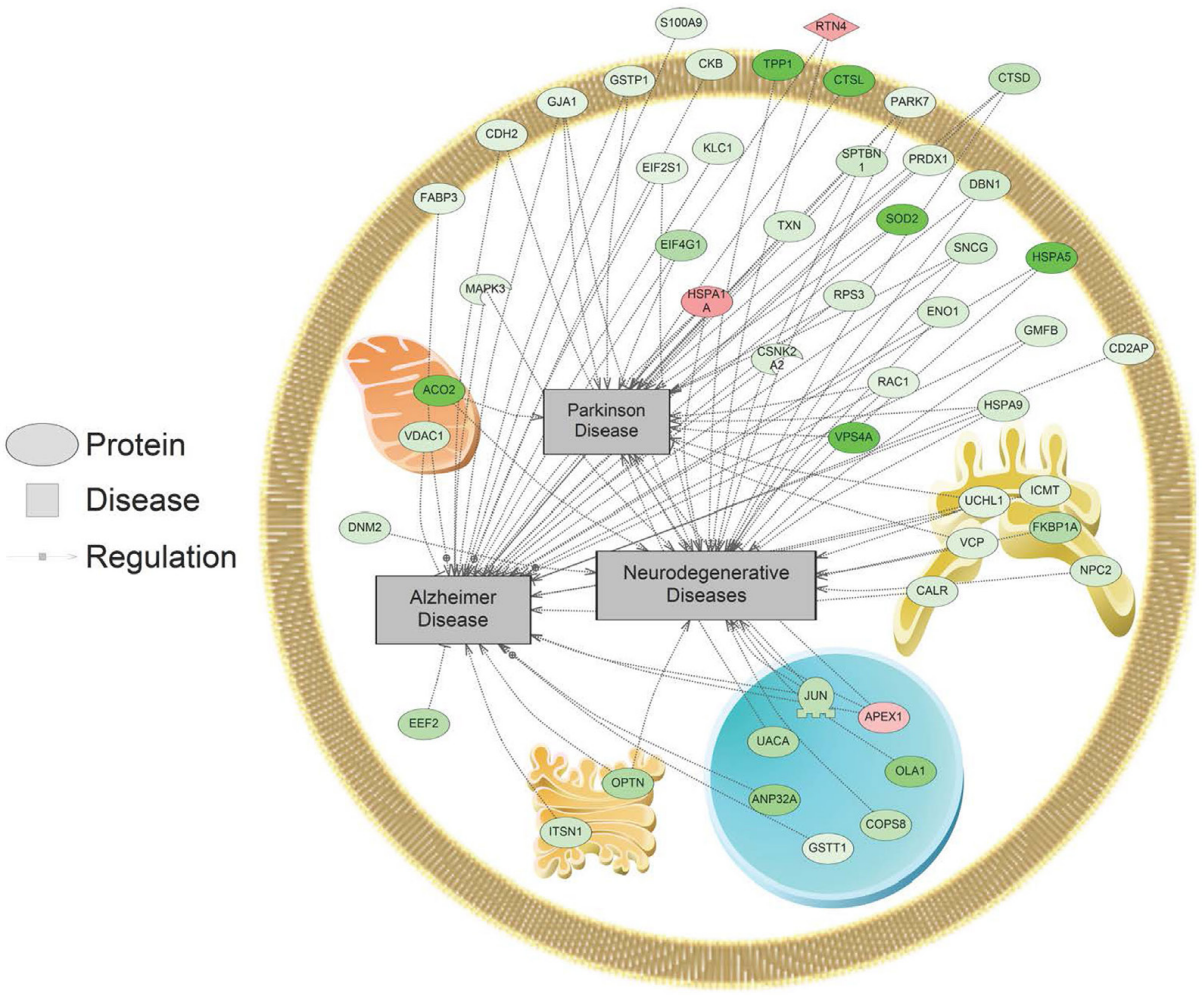
Alzheimer, Parkinson, and neurodegenerative disease pathways were enriched in primary *Carassius auratus* radial glial cell culture after 1,000 nM secretoneurin A treatment (*P* < 0.05) based on the protein profiles. Red indicates that the protein is increased, and green indicates that the protein is decreased in abundance relative to the control group. All abbreviations are provided in Table S4 in Supplementary Material.

In addition, SNEA was used to determine if regulated proteins were known to be associated with human diseases. Major themes in disease networks that were associated with proteins altered by 1,000 nM SNa included diseases of the CNS (brain injuries, Alzheimer disease, Parkinson disease, cerebral infraction, brain ischemia), diseases of the cardiovascular system (heart failure, cardiomyopathies, heart injury, cardiac hypertrophy), and neoplasms (lung, ovarian, colorectal, pancreatic, prostatic, breast, endometrial). An example of a network associated with Alzheimer, Parkinson, and neurodegenerative diseases is shown in Figure 8. All the identified subnetworks are presented in Table S3 in Supplementary Material.

## Discussion

This study used quantitative proteomics to determine the response of RGCs to SNa in an *in vitro* cell culture system. This effort to characterize the goldfish RGC proteome identified 1,363 unique proteins and adds to those previously reported (25). A total of 609 proteins showed changes in relative abundance across three concentrations of SNa, with higher doses eliciting a greater suppressive response in protein abundance. In each concentration, SNa regulated proteins that have a role in cell processes including actin organization, cell growth, proliferation, and migration. Previous research on the effects of SN in the CNS focused on neurons (26), so our work on the effects of SNa on the RGC transcriptome (15) and proteome (current study) is the first to characterize the effects of SNa in any glial cell type.

Assigning pathway ontologies to the identified proteins revealed many relationships to the immune system, including chemokine and cytokine signaling, T cell activation, TGFβ signaling, toll receptor signaling pathway, B cell activation, and interleukin signaling pathway. While the role of RGCs *per se* in neuroinflammation is not well studied, both the transcriptomic and proteomic data support the hypothesis that RGCs contribute to immune-related functions in fish. In the mammalian CNS, this is more typically associated with astrocytes, which possess both pro- and anti-inflammatory functions, depending on the mode of injury (27). In fish brain, RGCs are the main macroglia due to the lack of typical stellate astrocytes, thus RGCs likely share some functions with mammalian astrocytes in brain homeostasis (14). In addition, many neurotransmitter (5-HT, DA, ACh, glutamate, GABA, histamine) and hormone (thyrotrophin-release hormone, oxytocin, corticotropin-releasing factor) receptor-mediated pathways were identified, providing further evidence that neuronal–glial interactions are important in the control of this cell type. Our proteomic data show the presence of several well-characterized RGC markers including brain lipid binding protein (BLBP), glial fibrillary acidic protein (GFAP), tight junction protein ZO-1, Sox2, and vimentin (28, 29), confirming that these cultured cells are indeed RGCs. Furthermore, analysis of the differentially regulated proteins shows that SNa can regulate these classical protein markers of RGCs. At each concentration, SNa increased the expression of the intermediate filament protein vimentin. While vimentin is implicated in several glial processes, it has also been shown to be involved in the initial stages of neurite outgrowth in rat hippocampal neurons *in vitro* (30). However, the 1,000 nM SNa treatment decreased BLBP and ZO-1. These proteins are lost when RGCs differentiate into neurons. Moreover, SNa decreases the expression of aromatase B (*cyp19a1b*) in RGCs *in vivo* and *in vitro* in a dose-dependent manner (16). Taken together, these data suggest that SNa downregulates some of the genes related to the progenitor-like characteristics of this cell type.

A low dose of 10 nM SNa upregulated proteins known to be involved in processes of blood vessel development, actin organization, cytoskeleton organization and biogenesis, cell growth, migration, and proliferation. It has been previously established that SN can induce mouse angiogenesis and vasculogenesis (31, 32), which supports our data that 10 nM SNa increases the expression of proteins associated with blood vessel development. Mouse RGCs have been shown to regulate angiogenesis in the brain through their interactions with blood vessels (33). Since SNa can regulate proteins involved in blood vessel formation, this may control the function of RGCs to regulate angiogenesis. As a chemoattractant, SN has the ability to stimulate the migration of various cell types. SN can stimulate the migration of human monocytes both *in vivo* and *in vitro* at nanomolar concentrations (34). Similarly, SN induces eosinophil (35), dendritic cell (36), and fibroblast (37) chemotaxis. These studies indicate that the chemotactic activity of SN is dose dependent with maximal affects between 0.1 and 100 nM, and higher doses elicit an inhibitory affect. Interestingly, here, we report that proteins involved in cell migration are decreased by a high concentration of 1,000 nM SNa, indicating that SNa may regulate proteins involved in cell migration in a dose-dependent manner. In addition, both 100 and 1,000 nM concentrations regulated actin organization, cell growth, and cell proliferation protein networks; however, these higher concentrations decreased expression of proteins involved in these cell processes.

Following treatment with 1,000 nM SNa, subnetworks related to tight junction and gap junction assembly were enriched based on the proteins regulated. These cell processes were also identified by gene set enrichment analysis of transcriptomic data and were increased by SNa at the gene network level (15). Unlike the transcriptomic response, proteins involved in tight junction and gap junction assembly were downregulated, indicating that there is a complex regulation of the transcriptome and proteome that control these processes. Among the adherens junctions that were regulated by SNa, *N*-cadherin (cadherin 2) is a calcium-dependent adhesion molecule (38), and it is expressed to uphold RGC apical–basal polarity while anchoring adjacent RGCs to each other to create stem cell niches (39–42). *N*-cadherin controls mouse RGC function in neuronal migration and directing axon formation (42, 43). Furthermore, *N*-cadherin through its interactions with its effector β-catenin regulates RGC proliferation and differentiation (44–46). Reduction in mouse *N*-cadherin causes RGCs to migrate from the stem cell niche and stimulate neuronal differentiation (46). In our study, *N*-cadherin levels were increased at 10 nM and decreased at 1,000 nM SNa. Therefore, our data on *N*-cadherin expression indicate a role for SNa in the regulation of RGC-mediated neuronal migration, as well as RGC proliferation and differentiation.

Proteins involved in cellular processes such as neurite outgrowth and nerve fiber regeneration were also altered in abundance in RGCs following treatment with 1,000 nM SNa. The high dose of SNa significantly decreased the abundance of proteins known to be involved in neurite outgrowth. Although this cellular process was not enriched at lower doses, these data indicate that SNa has inhibitory effects on proteins that regulate neurite outgrowth. In contrast, SN stimulates neurite outgrowth in mouse cerebellar granule cells with a maximal concentration of 100 nM (47). Proteins related to nerve fiber regeneration were also decreased in goldfish RGCs exposed to 1,000 nM SNa. Previous findings show that SN treatment induces neural regeneration and neurogenesis in murine models of stroke (48). The data obtained from goldfish reveal protein networks that may underlie the role of SN in tissue repair and specifically in stem cell-like RGCs. SNa increased the expression of reticulon 4 (RTN4) in goldfish RGCs. RTN4 is represented in both the nerve fiber regeneration and the neurite outgrowth protein networks in goldfish RGCs. It is also known as a negative regulator of neurite plasticity in mammalian models by inhibiting neurite outgrowth and axonal regeneration (49–51). However, the major protein region of RTN4 that is involved in neurite growth inhibition is absent in zebrafish RTN4 (52) and is required for successful zebrafish axon regeneration (53). Therefore, given the opposite effects of RTN4 observed in zebrafish compared to mammals, future work should be directed toward elucidating the effects of SNa on neurite outgrowth and axon regeneration in fish through mechanisms involving RTN4.

Many proteome subnetworks were identified that correspond to diseases of the CNS and cardiovascular system along with several neoplasms. Protein networks in RGCs related to Alzheimer disease, Parkinson disease, brain ischemia, and brain injury were all regulated by 1,000 nM SNa. Changes in these networks are consistent with previous studies that have associated SN with these neuropathological conditions. SN is differentially regulated in brain ischemia (48, 54, 55), Alzheimer disease (56–58), and epilepsy (59, 60). It has been reported that circulating SN increases after heart failure and has a protective effect by reducing myocardial ischemia injury, cardiomyocyte apoptosis, and inducing angiogenesis (61, 62). Here, we show that SNa can regulate subnetworks related to cardiovascular diseases such as cardiomyopathies, heart injury, cardiomegaly, and heart failure. In addition, many subnetworks related to neoplasms were enriched after SNa treatment including lung, ovarian, colorectal, pancreatic, prostatic, breast, and endometrial. Importantly, SN has been shown to be expressed in several types of neuroendocrine tumors including prostate, lung, rectal, pancreatic, thyroid, duodenum, and appendix (63–65). Taken together, it is suggested that these networks underlie, in part, the molecular responses to SN that are mechanistically linked to these disease states.

The teleost brain has become a popular model for neurogenesis and neuroendocrinology because of the persistent abundance of progenitor RGCs in the adult brain and the high aromatase activity present in RGCs (29, 66, 67). Our research has implications in these fields as characterizing the RGC proteome can elucidate the molecular mechanisms that underlie these unique functions of fish RGCs. The proximity of SNa-immunoreactive magnocellular preoptic neurons with aromatase B/GFAP expressing RGCs prompted our efforts to characterize the effects of SNa on the transcriptome (15) and now the proteome of these multifunctional cells. For the first time, we show that the RGC proteome is responsive to nanomolar concentrations of SNa. Proteins regulated by SNa are implicated in many cell processes including those associated with blood vessel development, actin organization, cytoskeleton organization and biogenesis, neurite outgrowth, nerve fiber regeneration, cell growth, migration, and proliferation. At lower doses, SNa increased proteins involved in cell growth, migration, and proliferation, whereas higher doses of SNa downregulated proteins involved in these processes. This indicates that SNa has dose-dependent regulatory effects in RGCs. At 1,000 nM of SNa, proteins linked to the etiology of diseases of the CNS (brain injuries, Alzheimer disease, Parkinson disease, cerebral infraction, brain ischemia) were also differentially regulated. These data implicate that SNa in the control of pathways is important in cell proliferation and neurogenesis through direct actions on RGCs.

## Ethics Statement

The study was carried out in accordance with the Canadian Council on Animal Care and approved by the University of Ottawa animal care and veterinary services.

## Author Contributions

DD performed experiments, analyzed the data, and wrote the paper. CM performed experiments, analyzed the data, and contributed to writing the paper. LX performed experiments, analyzed the data, and contributed to writing the paper. VT helped design experiments and co-wrote the paper.

## Conflict of Interest Statement

The authors declare that the research was conducted in the absence of any commercial or financial relationships that could be construed as a potential conflict of interest.
